# 

**Published:** 1891-03-14

**Authors:** 


**Medical Women in England** - 343

**Passing Topics.**—The London County Council and Hospital Assessment - 344

**The Doctor's Armchair.**—The Gnat and the Camel - 345

**The History and Capabilities of Herbal Simples.** By W. T. Fernie, M.D. XXVIII.—The Dandelion - 346

**The Microscope in Medicine.** By Frank J. Wethered, M.D.—IX.—Examination of the Sputum (*continued*) - 347

**Notes and News** - 348

**General Charities** - 349

**Scraps and Gleanings** - 349

**Annotations.**—More Hospital Accommodation for North London—Sanitation at Huddersfield—A Question of Propriety - 350

**The Practitioner's Mirror and Retrospect:—**

Medicine.—Intestinal Antisepsis in Dyspepsia - 351

Surgery.—Typhlitis, Perityphlitis, and Appendicitis - 352

Ophthalmology.—Membranous Ophthalmia - 352

New Drugs, Appliances, and Things Medical.—Swinborne's Isinglass—The Sanitary and Antiseptic Respirator—"Angel-White" Toilet Powder - 353

The Practitioner's Bookshelf.—Rectal and Anal Surgery - 353

**The Lords' Committee** - 354

**Midwives Registration—A General Practitioner's Protest.** By W. D. Haslam, M.D. - 354

**The Student of Medicine.**—Leading Athletes—Appointments—Vacancies—Athletics—Students' Medical Societies.

EXTRA SUPPLEMENT—THE NURSING MIRROR.

**En Passant.**—Swansea Institute—Winchester District Work—Kingston Nursing Association—Breach of Promise—Fever Hospitals—Burton Nursing Institution—Charity at Cheltenham—Short Items—Cumberland Infirmary - cxxxi

**The Royal National Pension Fund for Nurses** - cxxxii

**Princess Christian's Daughter** - cxxxiii

**The Eastern Hospital Scandal** - cxxxiii

**A Night Experience at Kimberley Hospital** - cxxxiv

**The Nurse's Bookshelf**—Babyhood - cxxxv

**Everybody's Opinion.**—Paddington Green Children's Hospital—Personal Anecdotes - cxxxv

**Presentations** - cxxxv

**Rumples** - cxxxvi

**Notes and Queries** - cxxxvi

**Appointment** - cxxxvi

**Amusements and Relaxation** - cxxxvi

